# Cardiopulmonary bypass for total aortic arch replacement surgery: A review of three techniques

**DOI:** 10.3389/fcvm.2023.1109401

**Published:** 2023-03-30

**Authors:** Ying Cui, Xinhao Liu, Jiyue Xiong, Zhaoxia Tan, Lei Du, Jing Lin

**Affiliations:** Department of Anesthesiology, West China Hospital, Sichuan University, Chengdu, China

**Keywords:** type a aortic dissection (TAAD), aortic arch surgery, cardiopulmonary bypass (CBP), deep hypothermic circulatory arrest, perfusion technique

## Abstract

One treatment for acute type A aortic dissection is to replace the ascending aorta and aortic arch with a graft during circulatory arrest of the lower body, but this is associated with high mortality and morbidity. Maintaining the balance between oxygen supply and demand during circulatory arrest is the key to reducing morbidity and is the primary challenge during body perfusion. The aim of this review is to summarize current knowledge of body perfusion techniques and to predict future development of this field. We present three perfusion techniques based on deep hypothermic circulatory arrest (DHCA): DHCA alone, DHCA with selective cerebral perfusion, and DHCA with total body perfusion. DHCA was first developed to provide a clear surgical field, but it may contribute to stroke in 4%–15% of patients. Antegrade or retrograde cerebral perfusion can provide blood flow for the brain during circulatory arrest, and it is associated with much lower stroke incidence of 3%–9%. Antegrade cerebral perfusion may be better than retrograde perfusion during longer arrest. In theory, blood flow can be provided to all vital organs through total body perfusion, which can be implemented *via* either arterial or venous systems, or by combining retrograde inferior vena caval perfusion with antegrade cerebral perfusion. However, whether total body perfusion is better than other techniques require further investigation in large, multicenter studies. Current techniques for perfusion during circulatory arrest remain imperfect, and a technique that effectively perfuses the upper and lower body effectively during circulatory arrest is missing. Total body perfusion should be systematically compared against selective cerebral perfusion for improving outcomes after circulatory arrest.

## 1. Introduction

Most cases of acute type A aortic dissection (ATAAD) require surgical management. In the DeBakey classification, type I dissections originate in the ascending aorta and extend to at least the aortic arch, type II dissections involve the ascending aorta only (1). Hemiarch replacement surgery was performed when the intimal tear is localized along the ascending aorta or the lesser curvature of the transverse arch. In patients with an intimal tear localized along the greater curvature close to the supra-aortic vessels, total arch replacement surgery (TARS) was performed (2). Since its description in 1957, TARS has been performed, sometimes in combination with frozen elephant trunk (FET) implantation (3). Despite ongoing technical evolution (4), TARS remains a complex, challenging procedure. During FET implantation, a covered stent sutured to the distal end of a conventional tube graft is delivered antegrade to the distal aorta, where it is subsequently replaced. Using an FET means that only one anastomosis is required, greatly simplifying surgery in the next phase (5). The FET provides expansive radial force on the distal aorta and reduces the need for additional operations to manage false lumen, which may improve long-term survival (6). Circulatory arrest is required during TARS to maintain a clear surgical field that is important for proper antegrade placement of the FET and distal aortic anastomosis with the graft, reducing the risk of tearing when the distal aorta is cross-clamped.

The circulatory arrest technique block perfusion of the lower body and thereby cause ischemic injury, contributing to post-TARS mortality rates as high as 28% (7) as well as to rates of complications ranging from 0.8%–9% for stroke (8, 9), 17%–29% for neurological disorder (10), 6%–7% for respiratory failure (11, 12) and 19%–45% for acute kidney injury (13–15).

To attenuate ischemia during TARS, three techniques based on deep hypothermic circulatory arrest (DHCA) have been implemented. The first is DHCA alone, in which no perfusion is performed during arrest. The second is the combination of DHCA with antegrade cerebral perfusion (ACP) or retrograde cerebral perfusion (RCP), which may provide blood flow to the brain, but which still leaves the lower body at risk of ischemia. The third is the combination of DHCA with total body perfusion, which can be achieved through the arterial or venous systems, or through our recently proposed combination of ACP with retrograde inferior vena caval perfusion (RIVP) (16, 17).

Here we review these three approaches in order to guide future improvements in TARS.

## 2. Discussion

### 2.1. DHCA

DHCA involves complete circulatory arrest during anastomosis of the descending aorta and the graft. First used in the late 1950s (18), DHCA involves reducing systemic temperature to 18–20 °C. The primary goal during DHCA is to avoid cerebral ischemic injury because of the high oxygen requirements of the nervous system. Metabolism decreases by 5%–7% for each decrease of 1 °C in body temperature (19). As a result, cerebral oxygen consumption at 18 °C is only 12%–25% of the consumption at 37 °C. Hypothermia also protects the brain by promoting neuronal survival (20) and by inhibiting apoptosis (21), free radical production (22) and release of inflammatory cytokines (23) from astrocytes. In these ways, hypothermia decreases the release of excitatory neurotransmitters and protects vascular endothelial cells from ischemic injury (24).

By attenuating systemic hypoxic injury, DHCA prolongs the safe window for circulatory arrest from 5 min at 37 °C to 25 min at 18 °C, making TARS feasible (25). If DHCA alone lasts longer than 25 min, risk of neurological dysfunction increases significantly (26).

Unfortunately, neurological injuries are still prominent even during this safe window. Animal studies show that DHCA leads to elevation of S-100 protein, a marker of neurological injury (27), as well as neuronal apoptosis (28). When DHCA lasts up to 30 min, rate of stroke is 4%; 30–44 min, 7.5%; 45–59 min, more than 10%; 60–120 min or longer, as high as 14.6% (29). These rates may be even higher among patients older than 60 years (30).

DHCA leads to a lack of substrates for energy metabolism. It has been linked to substantial risk of postoperative morbidity and mortality, which can be attributed to dysregulation of cerebral blood flow (31) and coagulation, systemic inflammatory responses, requirement for massive blood transfusion (32), and requirement for long-term cardiopulmonary bypass during cooling and rewarming (33).

### 2.2. Selective cerebral perfusion

To attenuate cerebral ischemic injury, some cardiac centers proposed “selective cerebral perfusion” in the early 1980s, including antegrade cerebral perfusion (ACP) and retrograde cerebral perfusion (RCP).

#### 2.2.1. RCP

RCP is perfusion of oxygenated blood into the brain *via* the superior vena cava. It was first reported in the early 1980s (34), and it became widely used in TARS during the 1990s. RCP sends blood in a non-physiological manner, leading to concerns about whether it provides adequate blood supply to the brain. Animal studies have reported cerebral blood flow of only 0.02 ± 0.02 ml/min/100 g (35) or 0.5 ± 0.5 ml/min/100 g (36) under RCP, much lower than the 16 ± 7.7 ml/min/100 g provided by arterial perfusion. Increasing RCP pressure increases blood flow but also risk of cerebral edema. An RCP pressure of 25 mmHg may provide optimal balance of cerebral blood flow and cerebrospinal fluid pressure during deep hypothermia (20 °C) (37).

Studies suggest that RCP can protect the brain from ischemic injury better than DHCA (Table 1). One study of 207 patients undergoing aortic arch surgery with RCP showed rates of 30-day mortality of 10% and permanent neurological deficit of 6% (41). Another study of RCP reported a transient neurological deficit rate of 19%, permanent neurological deficit rate of 9%, and in-hospital mortality of 10% when core temperature was 14–20 °C during circulatory arrest (26). The superiority of RCP has also been supported in much larger studies. A retrospective analysis of 1,193 patients indicated slightly lower stroke rate with RCP than DHCA (2.8% vs. 4.2%) (39). A meta-analysis involving 26,968 patients found that relative to DHCA, RCP was associated with significantly lower operative mortality (OR 0.57, 95% CI 0.45–0.71) and postoperative stroke (OR 0.66, 95% CI 0.54–0.82) than DHCA, regardless of whether the arrest temperature was below or above 20 °C (40).

**Table 1:** Clinical studies comparing deep hypothermic circulatory arrest (DHCA) with retrograde cerebral perfusion (RCP).

Study	Publication year	Study design	Arms (n)	Mortality	Neurological events	Other outcomes
Coselli et al. (38)	1997	Retrospective	RCP (305), DHCA (204)	In-hospital mortality: 3.9% vs. 17.2% (significant)	Stroke: 2.6% vs. 6.4% (significant)	Not reported
Safi et al. (39)	2011	Retrospective	RCP (1002), DHCA (191)	Not reported	Stroke: Circulatory arrest within 40 min: 2.8% vs. 4.2% Circulatory arrest after 40 min: 1.7% vs. 30% (significant)	Not reported
Hameed et al. (40)	2019	Network meta-analysis	Total patients (26,968)	Operative mortality: OR 0.57, 95% CI 0.45-0.71 (RCP vs. DHCA)	Stroke: OR 0.66, 95% CI 0.54-0.82, TND: OR 1.27, 95% CI 0.93- 1.74	Myocardial infarction: OR 0.51, 95% CI 0.13-2.04, Respiratory complications: OR 0.99, 95% CI 0.72-1.35, Renal failure: OR 0.92, 95% CI 0.55-1.54

TND, transient neurological deficit.

However, RCP is not superior to DHCA in protecting vital organs other than the brain, reflected in the similar rates of the following complications for the two techniques: myocardial infarction (OR 0.51, 95% CI 0.13–2.04), respiratory complications (OR 0.99, 95% CI 0.72–1.35), and renal failure (OR 0.92, 95% CI 0.55–1.54) (40). Neither RCP nor DHCA perfuses the lower body may result in the similar rates of visceral complications.

#### 2.2.2. ACP

Uni- or bilateral ACP can be performed during circulatory arrest by inserting one or two catheters, respectively, into one or two of the following arteries: innominate, right subclavian, axillary, left/right common carotid, or radial. Another common approach is to use three catheters to cannulate three neck vessels from the inside. Several studies have shown ACP to be superior to DHCA in terms of postoperative mortality (OR 0.63, 95% CI 0.51–0.76) and cerebral infarction (OR 0.62, 95% CI, 0.51–0.75), but similar to DHCA in terms of transient neurological deficit (OR 1.03, 95% CI 0.78- 1.35), myocardial infarction (OR 1.38, 95% CI 0.56–3.39), respiratory complications (OR 0.89, 95% CI 0.67–1.18) and renal failure (OR 0.87, 95% CI 0.56–1.35) (40).

Whether uni- or bilateral ACP is more appropriate for a given patient should be decided carefully. In unilateral ACP, oxygenated blood reaches the contralateral cerebral hemisphere *via* the Willis circle. Bilateral ACP is recommended for patients showing insufficient blood flow in the contralateral hemisphere based on cerebral oxygen saturation, electroencephalography, transcranial Doppler and contralateral tympanic temperature (42). Nevertheless, some studies have suggested no significant differences between uni- or bilateral ACP in rates of mortality (7.6% vs. 9.8%, *P* = 0.19), transient neurological deficit (6.5% vs. 9.3%, *P* = 0.14) or permanent neurological deficit (5.8% vs. 6.9%, *P* = 0.53) among patients undergoing TARS (43, 44).

Patients undergoing unilateral ACP do appear to be at higher risk of mortality than those undergoing bilateral ACP during longer circulatory arrest (45). In one study, bilateral ACP was associated with significantly better overall survival than unilateral ACP among patients receiving perfusion longer than 50 min (46).

#### 2.2.3. ACP vs. RCP

Each of the two techniques offers advantages and disadvantages. ACP provides physiological blood flow to the brain, but it cannot remove debris or air embolisms from the perfused arteries, which may increase risk of stroke. RCP cannot perfuse the brain effectively because of venous shunts (33), so there is risk of brain edema, yet it does remove debris and air from arteries, it prevents micro-aggregation of blood cells, and it delays acidosis in the ischemic brain by removing metabolites. Therefore, RCP may be superior to ACP for patients with severe carotid stenosis, carotid dissection, or diffuse atheroma in the arch.

One animal study found similar intracranial pressure and S-100 levels in the ACP and RCP groups (29). However, the ACP showed higher blood glucose and lower glycerol levels, as well as a smaller proportion of apoptotic neurons in cerebral cortex. Numerous clinical studies have reported similar perioperative mortality rates for RCP and ACP (Table 2) (45–52). For example, one study of 8,169 patients with acute aortic dissection or ruptured aneurysm who underwent total arch replacement found no significant differences between ACP and RCP in 30-day mortality (3.2% vs. 4.0%, *P *= 0.247), hospital mortality (6.0% vs. 7.1%, *P *= 0.290), incidence of stroke (6.7% vs. 8.6%, *P *= 0.083), transient neurologic disorder (4.1% vs. 4.4%, *P *= 0.756), dialysis (3.9% vs. 3.8%, *P *= 0.828), or pneumonia (8% vs. 7.2%, *P *= 0.477) (48). While two studies suggested that RCP is associated with significantly higher rate of transient neurological deficit (OR 2.11, 95% CI 1.11–4.02) (48, 49), several studies (47, 50–52) and a meta-analysis (40) associated the two techniques with similar rates of permanent or transient neurological deficits.

**Table 2:** Clinical studies comparing retrograde cerebral perfusion (RCP) with antegrade cerebral perfusion (ACP).

Study	Publication year	Study design	Arms (*n*)	Mortality	Neurological events
Usui et al. (50)	1999	Retrospective	RCP (*n* = 75); vs. ACP (*n* = 91)	Operative mortality 21% vs. 24% 30-day mortality 13% vs. 16%	Neurologic dysfunction 19% vs. 16%
Okita et al. (48)	2001	Prospective	RCP (*n* = 30) vs. ACP (*n* = 30)	In-hospital mortality: 6.6% vs. 6.6%	TND: 33.3% vs. 13.3% Stroke: 3.3% vs. 6.6%
Milewski et al. (47)	2010	Retrospective	RCP/DHCA (*n* = 682) vs. ACP/MHCA (*n* = 94)	In-hospital mortality: 2.8% vs. 3.2% *p* = 0.753	PND: 2.8% vs. 3.2% TND 3.7% vs. 5.3%
Usui et al. (49)	2012	Retrospective	ACP (*n* = 2209) vs. RCP (*n* = 583)	30 days mortality: 2.6% vs. 3.5% Operative mortality: 4.1% VS 5.3%	TND: 5.8% vs. 3% Stroke; 3% vs. 5%
Misfeld et al (52)	2012	Retrospective	DHCA: (*n* = 220) vs. RCP: (*n* = 51) vs. UACP: (*n* = 123) vs. BACP: (*n* = 242)	30-day mortality 7.3% vs. 14.0% vs. 7.8% vs. 11.4%	PND 10.6% vs. 8.3% vs. 15.7% vs. 14.1%, TND 17.9% vs. 14.9% vs. 17.6% vs. 12.7% ACP vs. non-ACP: Stroke 9% vs. 15%
Di Mauro et al (45)	2013	Retrospective	DHCA (69) vs. ACP (189) vs. RCP (198)	Not reported	Neurological dysfunction: 17.4% vs. 14.6%. vs. 9.0% Stroke: 13.0% vs. 7.6% vs. 5.8% TND: 4.3% vs. 7.1% vs. 3.2%
Stamou et al (51)	2016	Retrospective	DHCA: (*n* = 184) vs. RCP: (*n* = 55) vs. ACP: (*n* = 84)	Perioperative mortality: 19% vs. 14.5% vs. 19.1% 5 years survival rate 48.8% vs. 61.8% vs. 66.8%	Stroke 14.3% vs. 21.8% vs. 14.1%

DHCA, deep hypothermic circulatory arrest; PND, permanent neurological deficit; TND, transient neurological deficit.

When combined with deep hypothermia, ACP may be safe for up to 80 min, whereas little evidence supports the safety of RCP beyond 50 min (53). Future work should explore whether these variable results reflect different durations of circulatory arrest.

#### 2.2.4. DHCA vs. moderate hypothermic circulatory arrest (MHCA)

Whether DHCA, defined as 14.1–20 °C (54), is superior to moderate hypothermia (MHCA), defined as 20.1–28 °C, remains controversial. Since ACP can fulfill the brain's requirements for 1 L/min/m^2^ of blood flow at 21–25 °C or 1–1.6 L/min/m^2^ at 28 °C (55), it does not require low circulatory temperatures. This may lead to better outcomes because lower temperature inhibits oxygen release from hemoglobin and lowers the activity of metabolic enzymes in surrounding tissue, which can delay recovery of neurological function. On the other hand, mild hypothermia (28.1–34 °C) may increase risk of severe ischemic injury (56, 57), although ACP under such conditions does improve cerebral blood flow for a limited time.

Numerous clinical studies have compared DHCA with MHCA (Table 3), and many have concluded that the two approaches are associated with similar mortality (51, 58–70). On the other hand, at least one study associated DHCA with significantly higher mortality, both in-hospital (8% vs. 1%) and at 30 days (9% vs. 2%) (67).

**Table 3:** Clinical studies comparing deep or medium hypothermic circulatory arrest in combination with selective cerebral perfusion.

Study	Publish year	Type of study	Temperature	Mortality	Neurological events	Other Outcomes
Harrington et al. (58)	2004	Prospective Study	DHCA 15 °C (*n* = 21) vs. MHCA + ACP 25 °C (*n* = 21)	4.8% vs. 14.2%	TND 4.8% vs. 23% Stroke 9.5% vs. 0%	Jugular bulb P_O2_ DHCA Decreased from 31.68 mmHg to 10 mmHg MHCA Unchanged from 24.8 mmHg to 25.57 mmHg Oxygen extraction DHCA: 1.22 to 2.95 ml/dl MHCA: 3.2 to 2.38 ml/dl
Zierer et al. (59)	2005	Retrospective study	DHCA + ACP/RCP 20-24 °C (*n* = 38) vs. MHCA + ACP 30 °C (*n* = 18)	15.8% vs. 5.5%	PND 13.2% vs. 5.5%, TND 13.2% vs. 11.1%	Re-exploration for bleeding: 36.8% vs. 16.7%
Halkos et al. (60)	2009	Retrospective study	DHCA 18 °C (*n* = 66) vs. MHCA + ACP 23.2 °C (*n* = 205)	23.1% vs. 4.3%	TND 4.8% vs. 1.5% PND 0% vs. 2.9%	Renal failure 15.4% vs. 5.7%
Milewski et al. (47)	2010	Retrospective Study	RCP/DHCA 21 °C *n* = 682 vs. ACP/MHCA 26 °C *n* = 94	2.8% vs. 3.2%	TND 3.7% vs. 5.3% PND 2.8% vs. 3.2%	Renal failure 4.5% vs. 5.3% Reoperation for bleeding 3.8% vs. 4.3%
Numata et al. (61)	2012	Retrospective study	DHCA <27.9 °C (*n* = 44) vs. MHCA >28 °C (*n* = 54)	6.1% vs. 6.1%	TND: 6.1% vs. 6.1% PND: 9.8% vs. 6.1%	Re-exploration 11% vs. 4.9% Acute renal failure 15% vs. 3.7%
Leshnower et al. (62)	2012	Retrospective study	Mild 28.5 °C (*n* = 277) vs. Moderate 24.3 °C (*n* = 223)	4.2% vs. 4.8%,	PND 7.2% vs. 2.5% TND 6.3% vs. 4.3%	Dialysis-dependent renal failure 4.1% vs. 4.0% Re-exploration 7.2% vs7.2%
Tsai et al.(63)	2013	Retrospective study	DHCA: 16.8 °C (*n* = 78) vs. MHCA: 22.9 °C (*n* = 143)	30-day mortality 9% vs. 2% in-hospital mortality 8% vs. 1%	Stroke 8% vs. 3%	Postoperative dialysis 3% vs. 2% Re-exploration for bleeding 3% vs. 7%
Tian et al. (64)	2013	Meta-analysis	DHCA (15–20 °C) (*n* = 813) vs. MHCA (22–25 °C) (*n* = 970)	13.5% vs. 11.1% OR: 1.39; 95%CI: 0.88-2.20	TND 8.0% vs. 10.3%, PND 12.8% vs. 7.3% Stoke 12.8% vs. 7.3%	Renal failure 13.3% vs. 12.6% Reoperation for bleeding 10.9% vs. 13.3%
Leshnower et al. (65)	2015	Retrospective study	DHCA 21.6 °C (*n* = 88) vs. MHCA 27.4 °C (*n* = 206)	14.36% vs. 9.2%	Stroke 8.5% vs. 8.3% TND 7.3% vs. 4.9%	Dialysis 12.2% vs. 7.3%
Vallabhajosyula et al. (66)	2015	Retrospective Study	DHCA<20 °C (*n* = 301) vs. MHCA 25-28 °C (*n* = 75)	1% vs. 1%	Stroke 0% vs. 2% Paralysis 1% vs. 0%	Renal failure 1% vs. 0% Reoperation for bleeding 3% vs. 5%
Arnaoutakis et al. (67)	2016	Retrospective study	DHCA 17.5 °C (*n* = 471) vs. MHCA 26.4 °C (*n* = 118)	0.9% vs. 0%	Stroke 1.7% vs. 1.7%	Acute kidney injury 14.3% vs. 16.2% Dialysis 0.8% vs. 0%
Fang et al. (68)	2019	Retrospective study	DHCA 14.1-20.0 °C (*n* = 340) vs. MHCA 20.1–28.0 °C (*n* = 287)	0.9% vs. 1.8%	Stroke 0.9 vs. 3.7%, (significant) Paraplegia 3.2% vs. 6.0%	Acute kidney injury 73.3% vs. 75.6% Renal replacement therapy 9.2% vs. 9.2% Reoperation 3.7% vs. 5.5%
Leshnower et al. (69)	2019	Randomized controlled trial	DHCA + RCP 14.1-20 °C (*n* = 11) vs. MHCA + ACP 20-28 °C (*n* = 9)	Mortality 0 vs. 0	Composite of stroke, transient ischemic attack 45% vs. 100%, (significant) Stroke 9% vs. 11% TND 0 vs. 22%	Acute kidney injury 0 vs. 0 Dialysis 0 vs. 0

DHCA, deep hypothermia circulatory arrest; MHCA, moderate hypothermia circulatory arrest; RCP, retrograde cerebral perfusion; ACP, antegrade cerebral perfusion; PND, permanent neurological deficit; TND, transient neurological deficit.

While several studies have reported that DHCA and MHCA are similar in rates of transient or permanent neurological deficits (47, 60, 61), meta-analyses suggest that the rate of permanent deficit may be higher for DHCA (64, 70). One of those meta-analyses also found the rate of stroke to be higher for DHCA (12.8% vs. 7.3%, *P* = 0.004) (64).

DHCA may be associated with higher rates of renal failure (60, 61), although several studies have reported similar rates of acute kidney injury and postoperative dialysis between DHCA and MHCA (51, 62, 63, 65–69). One meta-analysis concluded that DHCA was associated with greater risk of postoperative dialysis (70).

Given that MHCA allows lower temporary neurologic deficit, shorter CPB (70) and mechanical ventilation (61, 70), and intensive care unit stay, the available evidence supports the use of higher core temperatures with ACP in TARS.

### 2.3. Summary of selective cerebral perfusion techniques

TARS has progressed from involving DHCA to a combination of milder hypothermic arrest with selective cerebral perfusion *via* ACP or RCP. This has reduced rates of mortality and neurological complications, yet rates of organ dysfunction in the lower body remain high as a result of inadequate perfusion. For example, rates of spinal ischemia or hemiplegic paraplegia range from 4% to 25%, while rates of dialysis range from 6% to 18% (17). These adverse events likely become more frequent with longer circulatory arrest.

## 3. Total body perfusion

Three methods have been reported to improve lower-body perfusion while maintaining cerebral perfusion during circulatory arrest in TARS.

### 3.1. Perfusion *via* the abdominal aorta or femoral artery

In this approach, the proximal end of the descending aorta is often occluded with a balloon during circulatory arrest, and the lower body can be perfused *via* the femoral artery (71) or through a hollow balloon catheter (72). One study reported good protection of kidney and liver through lower-body balloon perfusion during distal anastomosis in aortic arch repair; a flow rate of 32.9 ± 7.5 ml/kg/min provided blood pressure of 44.1 ± 12.5 mmHg in the lower extremities (72). A study of thoracic or thoracoabdominal aortic repair with balloon deployment reported rates of overall 30-day mortality of 4.75%, renal failure of 4.75%, heart failure of 9.5% and pulmonary complications of 29%, with no incidence of spinal cord neurological deficit or stroke (73). Multivariate analysis in a third study linked aortic balloon occlusion to lower risk of renal and hepatic injury, faster postoperative recovery of consciousness, and reduced need for perioperative red blood cell transfusion (74).

This method is safe for patients with undissected aneurysms, but it may increase risk in those with ATAAD. First, balloon occlusion may cause intimal injury of the descending aorta, which may be aggravated by longer occlusion (75) and atherosclerosis. Second, poor sealing of the balloon may obscure the surgical field and thus increase surgical risk. Third, perfusion through the femoral artery may exacerbate dissection if the inlet has been closed with a rigid elephant trunk but the outlet under the trunk is open.

### 3.2. Total body retrograde perfusion

Total body retrograde perfusion (TBRP), which involves perfusing the entire body in a retrograde manner, can be achieved, in theory, *via* both the superior and inferior venae cavae, because the cerebral and visceral organs lack a venous valve. However, we are aware of only two animal studies (76, 77) and one case report (78) that have reported such perfusion. A case series of two patients from our medical center described initiation on TBRP but conversion to RCP after only a few minutes because of inadequate blood flow (79).

A recent variation of TBRP, which could be called “brain-first TBRP”, is a modified form of RCP (80). It provides blood flow first to the brain, then to the lower body *via* communicating branches, such as the azygos vein, between the superior and inferior venae cavae. In this approach, both superior and inferior venae cavae are tethered with bands around the cannula, and the distal ends of the drainage tubes in both venae cavae are clamped. In the report of this approach, retrograde blood flow was observed in kidney and liver, inferior vena caval pressure was maintained 11 ± 3 mmHg and central venous pressure was maintained at 21 ± 2 mmHg, without fluid retention.

TBRP remains, for the moment, entirely experimental, but it may be worth examining in future studies.

### 3.3. Combination of retrograde inferior vena caval perfusion (RIVP) with ACP

**Figure 1:**
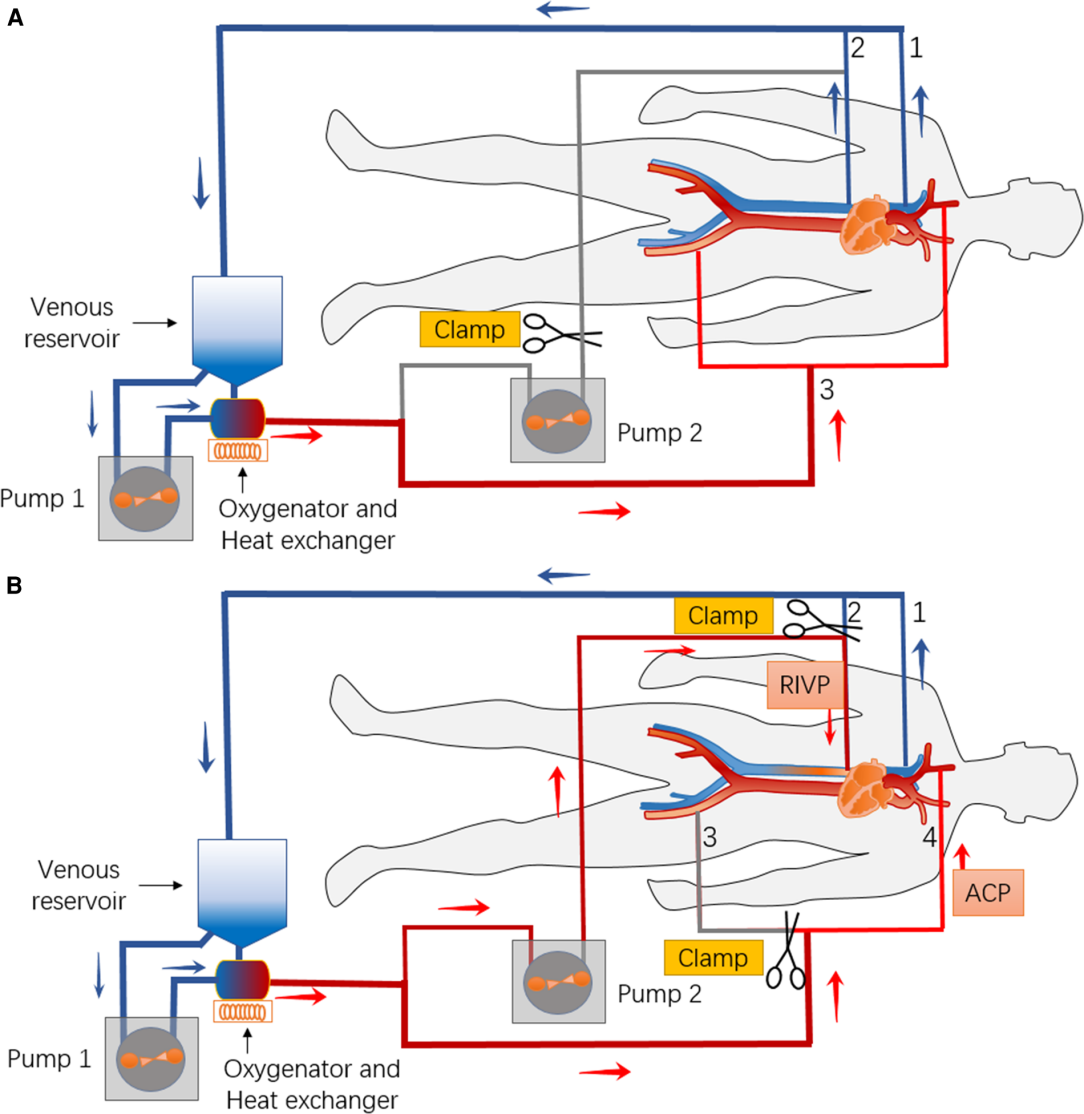
Schematic of the bypass circuit and its connection with the body for combination of retrograde inferior vena caval perfusion (RIVP) with antegrade cerebral perfusion (ACP). (**A**) Before and after combination of RIVP and ACP, venous blood from the superior vena cava *(1)* and inferior vena cava *(2)* is returned to the reservoir and driven by pump 1 to the oxygenator, then back to the body (femoral artery, aorta, innominate artery.) *(3)*. (**B**) During RIVP and ACP, venous blood from the superior vena cava *(1)* is returned to the reservoir and driven by pump 1 to the oxygenator. Part of the oxygenated blood is routed for ACP *(4),* while the other part is driven by pump 2 back to the lower body *via* the inferior vena cava *(2)*.

We speculated that RIVP could provide blood flow to the low body analogously to retrograde cerebral perfusion, leading us to propose the combination of RIVP with ACP during circulatory arrest. To test this idea, we used two pumps to drive blood to the brain and viscerae separately at different perfusion pressures (Figure 1) (16). ACP was maintained at a pressure of 40-60 mmHg and flow rate of 5–12 ml/kg/min. RIVP was maintained at a venous pressure below 25 mmHg, with a blood flow of 9.12 ± 3.01 ml/kg/min. Reversed blood flow in the liver and kidney during RIVP was observed by transesophageal echocardiogram. Blood gas analysis of distal aortic drainage showed the oxygen partial pressure to be 50-80 mmHg (unpublished data), much lower than the 200-300 mmHg in RIVP blood. This indicates that oxygen in RIVP blood was absorbed by the lower body.

A pilot study at our medical center involving 76 TARS patients compared outcomes between those who underwent ACP alone or in combination with RIVP (81). Both groups showed similar rates of the primary aggregate outcome (42% vs. 61%, OR 0.60, 95% CI 0.21–1.62, *P *= 0.31), which included paraplegia, postoperative dialysis-dependent renal failure, severe liver dysfunction, gastrointestinal complications, and all-cause mortality. However, ACP + RIVP was associated with lower incidence of transient neurological deficits (26% vs. 58%, OR 0.26, 95% CI 0.10–0.67, *P *= 0.006), shorter intubation duration (25 h vs. 47 h, *P *= 0.022) and smaller consumption of blood products. Similar results were obtained from interim analysis of more than 200 patients in an ongoing randomized controlled trial at five heart centers in China (unpublished data).

Before RIVP can be widely implemented in the clinic, several questions should be addressed. The association between pressure and blood flow during RIVP under different temperatures should be investigated. The optimal temperature for circulatory arrest should be determined, and whether RIVP can provide adequate oxygen to vital organs should be verified, in particular to the gastrointestinal tract, spinal cord, liver, and kidneys.

## 4. Conclusion

TARS was first carried out with DHCA, which led to high mortality and morbidity. The use of selective cerebral perfusion and higher body temperatures significantly reduced these adverse outcomes, but transient or permanent neurological defects as well as dysfunction of vital organs in the low body remain frequent. Maintaining total body perfusion during circulatory arrest, such as by combining RIVP and ACP, may be even better than selective cerebral perfusion, but this approach should be tested rigorously and systematically in diverse patient populations.
